# Complete mitogenome of Hainan medaka *Oryzias curvinotus* (Teleostei: Beloniformes) and transcriptional differences between male and female liver

**DOI:** 10.1080/23802359.2017.1303340

**Published:** 2017-03-21

**Authors:** Zhongduo Wang, Shuisheng Long, Jian Liao, Chengqin Huang, Hairui Zhang, Shunkai Huang, Yanping Zhang, Li Liu, Yusong Guo

**Affiliations:** Key Laboratory of Aquaculture in South China Sea for Aquatic Economic Animal of Guangdong higher Education Institutes, Fisheries College, Guangdong Ocean University, Zhanjiang, China

**Keywords:** *Oryzias curvinotus*, Hainan medaka, mitogenome, RNA-Seq, phylogenetic analysis

## Abstract

In this study, combining the liver transcripts from both sexes by RNA-Seq with DNA sequences by conventional PCRs, we have determined the complete mitogenome of Hainan medaka *Oryzias curvinotus* collected from the mangrove seawater of the Leizhou Peninsula in tropical South China (Accession no.: KY364884). The mitochondrial genome is 16,676 bp, and its content and structure are highly homologous to those of other teleostean fishes, including 13 protein-coding genes (PCGs), 2 rRNAs, 22 tRNA and 1 control region. Among the PCGs, ATG is used as the initiation codon, except for GTG in COI gene. There are 7 overlapping genes with overlap lengths ranging from 1 to 10 nucleotides (nt), while ten intergenic regions with a total of 66 nt and a maximum interval of 37 nt between tRNA^Asn^ and tRNA^Cys^. Moreover, the data from RNA-Seq shows that the significant differences exist in the expression patterns of mitogenomes between male and female.

Hainan medaka *Oryzias curvinotus* (Teleostei: Beloniformes) is a potential model fish akin to the freshwater Japanese medaka (*Oryzias latipes*), mainly distributed along the northwest coast of the South China Sea (Parenti 2012; Ta & Tran 2016). However, the mitochondrial genome of *O. curvinotus* was still not determined.

Here, the mature fishes were collected from National Mangrove Nature Reserve in Leizhou Peninsula, Guangdong Province, China. The muscle was used to extract DNA (Guo et al. 2016), while the livers of females and males were used to purify total RNA for transcriptomic sequencing (RNA-Seq) (Wang et al. 2009). The typical specimen and DNA were deposited in the Guangdong Ocean University. According to the sequences of RNA-Seq, three pairs of primers for conventional PCR were designed. The amplified DNA products were sequenced to fill the gaps and substitute the 3′ transcript ends between the Cytb gene and the control region, the control region and the 12s rRNA gene, the 12S rRNA gene and the 16S rRNA gene.

As a result, we have determined the mitogenome of *Oryzias curvinotus*. Based on the complete mtDNA, as shown in Figure 1, the phylogenetic trees supported the genetic relationship between the *O. curvinotus* and the *O. luzonensis* is the closest among the 7 species of genus *Oryzias*: *O. curvinotus, O. luzonensis*, *O. latipes*, *O. minutillus*, *O. javanicus*, *O. sinensis* and *O. melastigma* (also *O. dancena*), which is consistent with the previous reports using the nuclear tyrosinase and mitochondrial 12S and 16S rRNA genes (Naruse 1996; Takehana et al. 2005).

**Figure 1.**
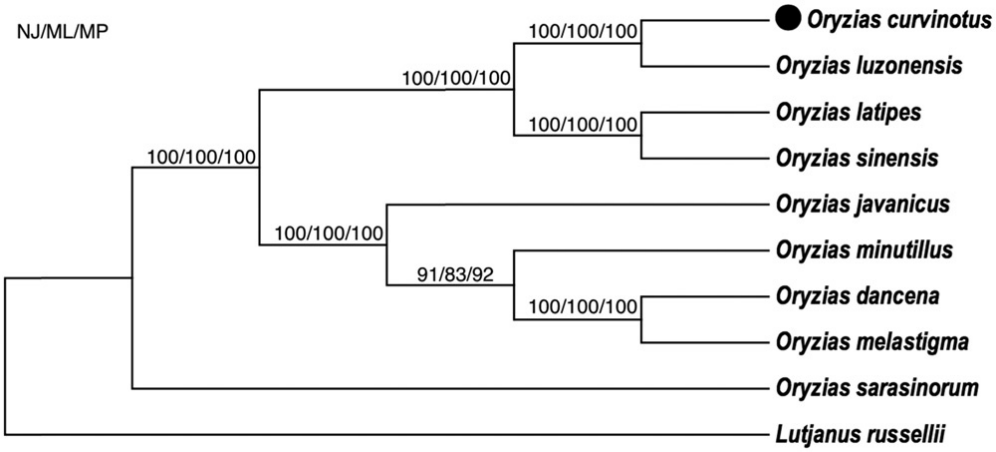
Neighbor-joining (NJ), maximum-likelihood (ML) and maximum-parsimony (MP) consensus tree based on mitogenomic DNA sequences. All the bootstrap values are indicated at the nodes.

Furthermore, through mapping the clustered transcripts to the annotated mitogenome of *O. curvinotus*, the obvious differences of the transcript units and their expression levels were observed between both sexes. Within a sex, the expression levels were also various among the transcripts. Torres et al. (2009) reported a similar result found in *drosophila*. Recently, more researches indicated that mitogenomic transcripts should be more complicated processing than previously assumed (Wang et al. 2013; Perera et al. 2016).
